# Symptomatic orthostatic hypotension due to standing mid-left ventricular obstruction: a case report

**DOI:** 10.1093/ehjcr/ytae566

**Published:** 2024-10-23

**Authors:** Leopoldo Ordine, Maria Angela Losi, Grazia Canciello, Felice Borrelli, Giovanni Esposito

**Affiliations:** Department of Advanced Biomedical Sciences, University Federico II, via S.Pansini, 5, I-80131 Naples, Italy; Department of Advanced Biomedical Sciences, University Federico II, via S.Pansini, 5, I-80131 Naples, Italy; Department of Advanced Biomedical Sciences, University Federico II, via S.Pansini, 5, I-80131 Naples, Italy; Department of Advanced Biomedical Sciences, University Federico II, via S.Pansini, 5, I-80131 Naples, Italy; Department of Advanced Biomedical Sciences, University Federico II, via S.Pansini, 5, I-80131 Naples, Italy

**Keywords:** Orthostatic hypotension, Restrictive hypertrophic cardiomyopathy, Mid-left ventricular obstruction, Syncope, Case report

## Abstract

**Background:**

Orthostatic hypotension (OH) is a common cardiovascular disorder typically associated with autonomic dysfunction. However, various other mechanisms can contribute to its occurrence.

**Case summary:**

An 88-year-old woman was referred to the cardiology unit due to recurrent syncope episodes while standing. Echocardiography revealed a normally contracting left ventricle with severe hypertrophy, a restrictive filling pattern, reduced stroke volume, and a decreased inferior vena cava diameter (4 mm/m²). In the standing position, she experienced syncope, and invasive blood pressure monitoring confirmed OH, alongside a normal increase in heart rate and evidence of mid-left ventricular obstruction (MVO) on echocardiogram. Discontinuation of diuretics and administration of fluids and beta-blockers effectively resolved the OH.

**Discussion:**

This case underscores the importance of considering mechanisms beyond autonomic dysfunction and volume depletion in the aetiology of OH in elderly patients. Notably, this is the first documented case of OH associated with MVO occurring in an upright posture, resulting in a significant decrease in cardiac output and subsequent syncope. Preventing volume depletion and using non-vasodilating beta-blockers may represent optimal therapeutic strategies in such cases.

**Learning points**

- To underscore the pathophysiological mechanisms of the development of orthostatic hypotension
- To understand the role of mid-left ventricular obstruction in developing orthostatic syncope

## Introduction

In healthy individuals, transitioning from a reclined to an upright position typically results in a decrease in cardiac output, offset by an increase in adrenergic response which boosts heart rate (HR), myocardial contractility, and peripheral vasoconstriction. In the elderly, dysautonomia can impair compensatory mechanisms, leading to orthostatic hypotension (OH) and orthostatic syncope. However, various other mechanisms may contribute to OH, with many cases remaining idiopathic (*Table 1*).^1,2^ Here, we present a case of an elderly patient with recurrent orthostatic syncope despite normal autonomic function.

**Table 1:** Aetiology of OH

Drug-induced OH	Diuretics (thiazide and loop); vasodilators (nitrates, calcium channel blockers); central antihypertensives (clonidine); sedatives (phenothiazine); antipsychotics (olanzapine); tricyclic antidepressant (amitriptyline); α-blockers (doxazosin); phosphodiesterase inhibitors (sildenafil); Parkinson’s drugs (levodopa); alcohol
Volume depletion	Haemorrhage, diarrhoea, vomiting, etc.
Primary autonomic failure	Pure autonomic failure; multiple system atrophy; Parkinson’s disease
Secondary autonomic failure	Elderly; diabetes; amyloidosis; kidney failure; spinal cord injuries; auto-immune autonomic neuropathy; paraneoplastic autonomic neuropathy

## Summary figure

**Figure:**
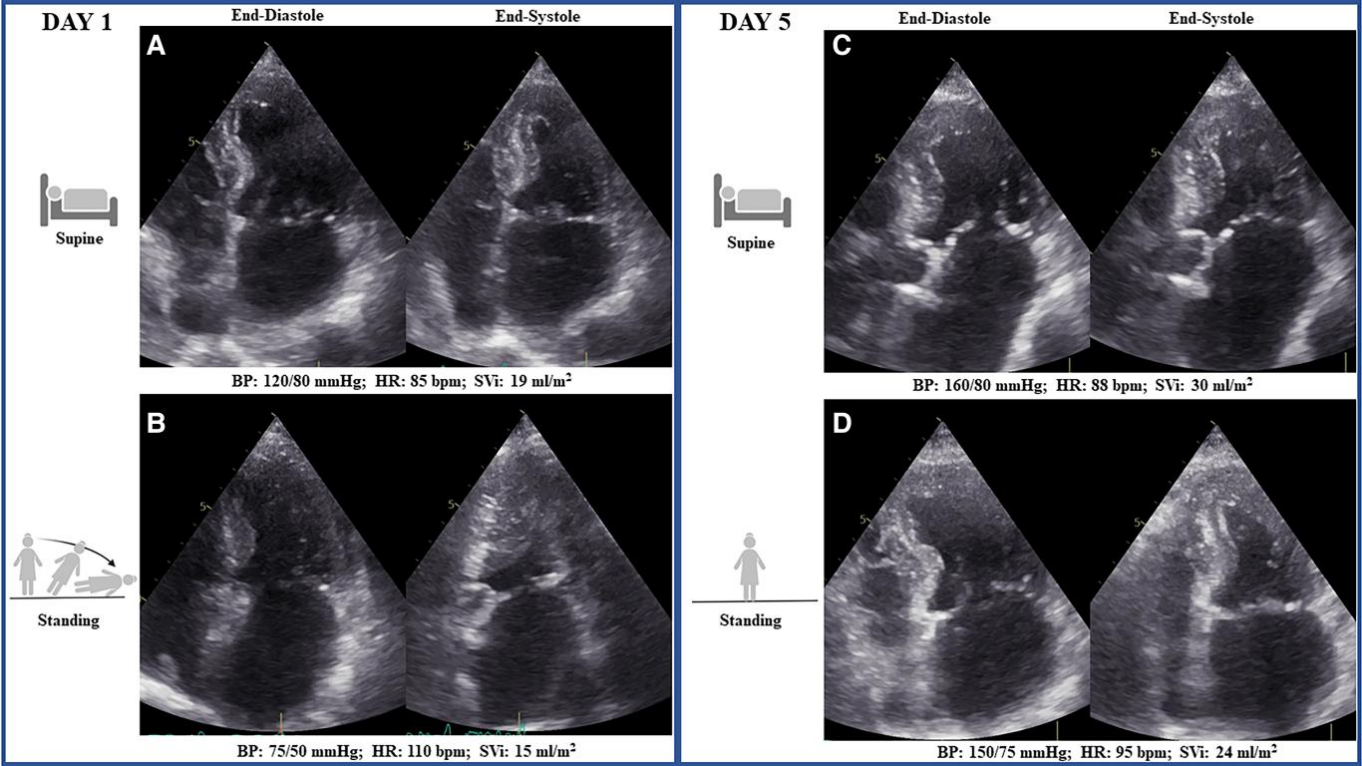
(*A*) At baseline, an echocardiogram in the supine position showed a non-dilated, hypertrophied, small left ventricle with restrictive physiology and reduced stroke volume index (SVi). (*B*) While standing, hypotension and symptoms developed with echocardiographic evidence of mid-left ventricular obstruction (MVO). (*C*) On the fifth day in the supine position, SVi was increased. (*D*) In the standing position, MVO was not present any further. BP, blood pressure; HR, heart rate; SVi, stroke volume index; MVO, mid-left ventricular obstruction.

## Case presentation

**Figure 1:**
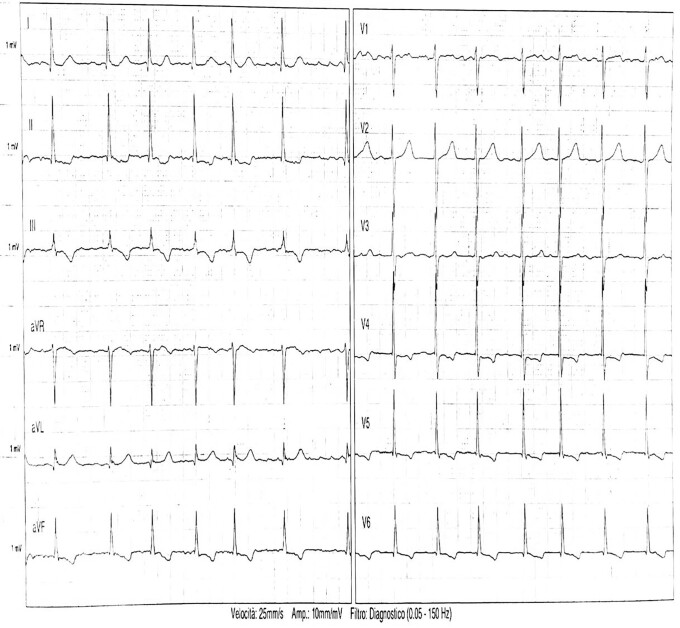
Electrocardiogram conducted upon admission which showed atrial fibrillation and diffuse repolarization abnormality.

An 88-year-old woman with a history of hypertension, dyslipidaemia, permanent atrial fibrillation (AF), mild carotid atherosclerotic disease, and Stage 3a chronic kidney, with no family history of cardiomyopathy or sudden cardiac death, was referred to our cardiology unit for recurrent unexplained episodes of orthostatic syncope. She had been experiencing daily syncope episodes over the past 2 months, with her most recent incident resulting in a facial fracture. Upon admission, she showed no signs of heart failure but reported mild shortness of breath with moderate exertion [New York Heart Association (NYHA II)]. The physical examination revealed a 2/6 systolic murmur in the aortic area, bilateral fine crackles at the lung bases, and no lower limb oedema. She weighed 60 kg and was 160 cm tall (body mass index: 23.44 kg/m^2^; body surface area: 1.62). Her daily medications included bisoprolol 1.25 mg, furosemide 25 mg, spironolactone 50 mg, and apixaban 5 mg. Laboratory tests revealed an elevated creatinine level (1.55 mg/dL), mild normochromic and normocytic anaemia (haemoglobin: 10.9 g/dL), and an increased N-terminal-pro B-type natriuretic peptide (NT-proBNP) level of 3621 pg/mL. The electrocardiogram (ECG) confirmed AF with a mean ventricular HR of 85 b.p.m. (*Figure 1*), and continuous ECG monitoring showed no brady- or tachyarrhythmia episodes. Invasive blood pressure (BP) monitoring showed an average supine BP of 135/70 mmHg.

A transthoracic echocardiogram, performed in the supine position (*Summary figure A*; Supplementary material online, *Video S1*), revealed left and right atrial enlargement, a restrictive left ventricular (LV) filling pattern, a small LV dimension, LV hypertrophy with maximal wall thickness (MWT) at the mid-anterior septum of 16 mm, and an LV ejection fraction (EF) of 63%, albeit with a low SVi of 19 mL/m^2^. The inferior vena cava was of small calibre (4 mm/m^2^) (*Table 2*).

**Table 2:** Echocardiographic findings on Days 1 and 5, both in supine and standing position

	Day 1 Supine position	Day 1 Standing position	Day 5 Supine position	Day 5 Standing position	Normal ranges^a^
LA volume (mL)	102		96		22–52
LA volume/BSA (mL/m^2^)	64		60		16–34
LA AP (mm)	51		50		27–38
E wave velocity (m/s)	1.1		1		0.5–1.0
E wave deceleration time (ms)	115		120		150–200
IVSd (mm)	16		16		6–9
LVIDd (mm)	37		38		38–52
LVIDs (mm)	26		27		22–35
PWd (mm)	10		10		6–9
RWT	0.54		0.53		<0.42
LV mass (g)	167		173		66–150
LV mass/BSA (g/m^2^)	103		107		44–88
LVEDV (mL)	49	27	68	53	46–106
LVEDV/BSA (mL/m^2^)	30	17	42	33	29–61
LVESV (mL)	18	3	20	15	14–42
LVESV/BSA (mL/m^2^)	11	2	12	9	8–24
LV EF (%)	63	88	71	72	54–74
SVi (mL/m^2^)	19	15	30	24	35–65
AoSV (mm)	33		33		30 ± 0.3
AoPxA (mm)	35		35		27 ± 0.4
Vmax AoV (m/s)	2.54		2.25		1.0–1.7
MaxPG AoV (mmHg)	26		23		<10
MeanPG AoV (mmHg)	16		14		<5
IVC (mm)	7		14		<21
IVC/BSA (mm/m^2^)	4		9		8–11,5
TAPSE (mm)	18		19		≥17
TR Vmax (m/s)	3.04		2.8		<2.8
TR max PG (mmHg)	37		32		<30
mPAP (mmHg)	42		37		<25

BSA, body surface area; LA, left atrium; LA AP, left atrium anteroposterior diameter; LV, left ventricular; EDV, end-diastolic volume; ESV, end-systolic volume; EF, ejection fraction; IVSd, intraventricular septum diastole; LVIDd, left ventricular internal dimension diastole; LVIDs, left ventricular internal dimension systole; PWd, posterior wall diastole; RWT, relative wall thickness; SVi, stroke volume index; AoSV, aorta sinuses of Valsalva; AoPxA, aorta proximal ascending; Vmax AoV, aortic valve max velocity; MaxPG AoV, aortic valve max peak gradient; MeanPG Ao, aortic valve mean peak gradient; TAPSE, tricuspid annular plane systolic excursion; TR Vmax, tricuspid regurgitation max velocity; TR max PG, tricuspid regurgitation max peak gradient; mPAP, mean pulmonary arterial pressure; IVC, inferior vena cava.

^a^The normal ranges refer to female subjects.

The patient was then invited to assume the standing position: BP dropped to 75/50 mmHg within 1 min, HR increased to 110 b.p.m., and the patient became symptomatic. During the same minute also, an echocardiogram was performed, showing an increase in LV EF to 88%, and MVO, with a further decrease in SVi (15 mL/m^2^) (*Summary figure B*; *Table 2*; Supplementary material online, *Video S2*).

Based on the clinical and echocardiographic findings, diuretics were discontinued, and fluid intake was started. In addition, bisoprolol 1.25 mg was replaced by metoprolol 100 mg. After 4 days, supine BP rose to 160/80 mmHg with an HR of 88 b.p.m., without evidence of OH (orthostatic BP 150/75 mmHg and HR 95 b.p.m.). The echocardiogram (*Table 2*) showed increased SVi in the supine position (30 mL/m^2^) with a stable LV EF (71%) (*Summary figure C*). In the standing position, there was no further evidence of MVO, with SVi of 24 mL/m^2^ and LV EF of 72% (*Summary figure D*). The patient was discharged with metoprolol, low-dose diuretics, and anticoagulant therapy.

During the follow-up visits at 1 and 3 months, the patient remained clinically stable (NYHA II) without evidence of OH, and no further orthostatic syncopal episodes were reported. The echocardiographic control confirmed the absence of MVO in the standing position. The patient was screened for cardiac amyloidosis with normal results in monoclonal protein testing (serum and urine immunofixation and serum kappa/lambda free light chain ratio) and a negative bone scintigraphy (Perugini score grade I). Cardiac magnetic resonance was not possible due to claustrophobia. Genetic testing has not yet been performed.

## Discussion

Orthostatic hypotension can be categorized into classic OH, where there is a sustained drop in systolic BP of at least 20 mmHg and/or diastolic BP of at least 10 mmHg within 3 min of assuming an upright position; delayed OH, involving a gradual reduction in BP from 3 to 45 min; and initial OH, as in our case, characterized by a sudden drop in BP (more than 40 mmHg in systolic BP and/or more than 20 mmHg in diastolic BP) within 30 s of standing.^1^ When standing, the sympathetic nervous system compensates for the reduced venous return by increasing vascular tone and utilizing chronotropic and inotropic effects. In elderly patients, OH is often attributed to the natural decline in baroreceptor sensitivity.^2,3^ Our case stands out because the HR increased correctly in the upright position, indicating that OH was not caused by dysautonomia. In addition, the clinical characteristics of OH in our patient exclude orthostatic vasovagal syncope, often seen after prolonged standing, and postural orthostatic tachycardia syndrome, which mainly affects young women and is marked by an HR increase (>30 b.p.m.) upon standing, without a simultaneous drop in BP.^1,2^

In our patient, despite a normal increase in HR upon standing, there was no corresponding increase in SVi. This phenomenon can be attributed to several factors. The presence of restrictive pathophysiology suggests that a sufficient LV volume is necessary to overcome high LV filling pressure, thus ensuring adequate LV filling.^4,5^ According to the European Society of Cardiology, in this condition, stroke volume remains fixed and only increases with elevated HR.^6^ In our patient, dehydration resulted in reduced LV filling volume which further decreased in the standing position despite an increase in HR. This case supports the link between LV restrictive pattern and decreased cardiac output during tilt testing in hypertrophic cardiomyopathy patients, highlighting how orthostatic positioning can induce syncope due to elevated LV filling pressure causing a critical reduction in cardiac output.^7^ This phenomenon is also observed in up to 50% of haemodialysis patients, where factors such as hypovolaemia or high myocardial stiffness may lead to restrictive filling and orthostatic syncope.^8^

Another contributing factor to OH-induced syncope was MVO which was diagnosed because of LV systolic obliteration. Although we were unable to detect an intraventricular pressure gradient, it is important to note that Doppler studies may not always identify mid-LV gradients due to the cessation of flow at this level^9,10^ (see Supplementary material online, *Figures S1* and *S2*).

**Figure 2:**
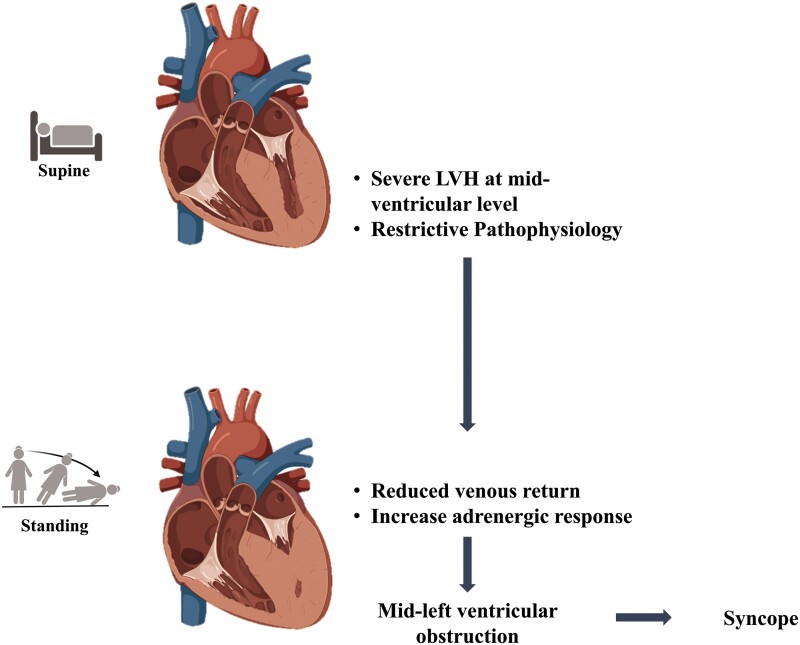
The pathophysiological mechanism involved in the development of mid-left ventricular obstruction. LVH, left ventricular hypertrophy.

We hypothesize that both anatomical and pathophysiological factors contribute to MVO (*Figure 2*). Anatomical factors include severe LV hypertrophy at the mid-ventricular level and changes in LV shape deformation when standing. This deformation causes MVO in a standing position, due to pathophysiological factors such as an adrenergic response that induces hypercontractility of the left ventricle and hypovolaemia that exacerbates underfilling of the left ventricle.^11^ Correct identification of OH and syncope mechanism guided precise treatment: discontinuation of diuretics, intravenous fluids to increase filling pressures, and non-vasodilating beta-blockers like metoprolol^12^ to enhance diastolic ventricular filling time and decrease inotropy. These interventions improved haemodynamics, resolving symptoms. Understanding the underlying mechanism of OH is crucial, especially considering MVO could be part of the aetiology.

## Lead author biography


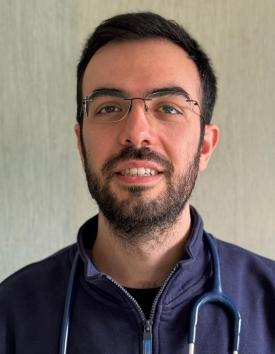
Leopoldo Ordine is a cardiology resident at the University Federico II in Naples, Italy. He has a keen interest in pursuing a career in cardiology, particularly in the areas of cardiomyopathy and cardiovascular imaging.

## Supplementary Material

ytae566_Supplementary_Data

## Data Availability

The data underlying this article are available in the article and in its online supplementary material.

## Supplementary material

**Consent:** The authors confirm that informed written consent was obtained before the publication of this case report in line with the COPE guidelines.

**Funding:** None declared.
