# Supplementary figures and images for: Symptomatic orthostatic hypotension due to standing mid-left ventricular obstruction: a case report
Source: Eur Heart J Case Rep. 2024 Oct 23;8(11):ytae566. doi: 10.1093/ehjcr/ytae566 (PMC11538991; doi:10.1093/ehjcr/ytae566)

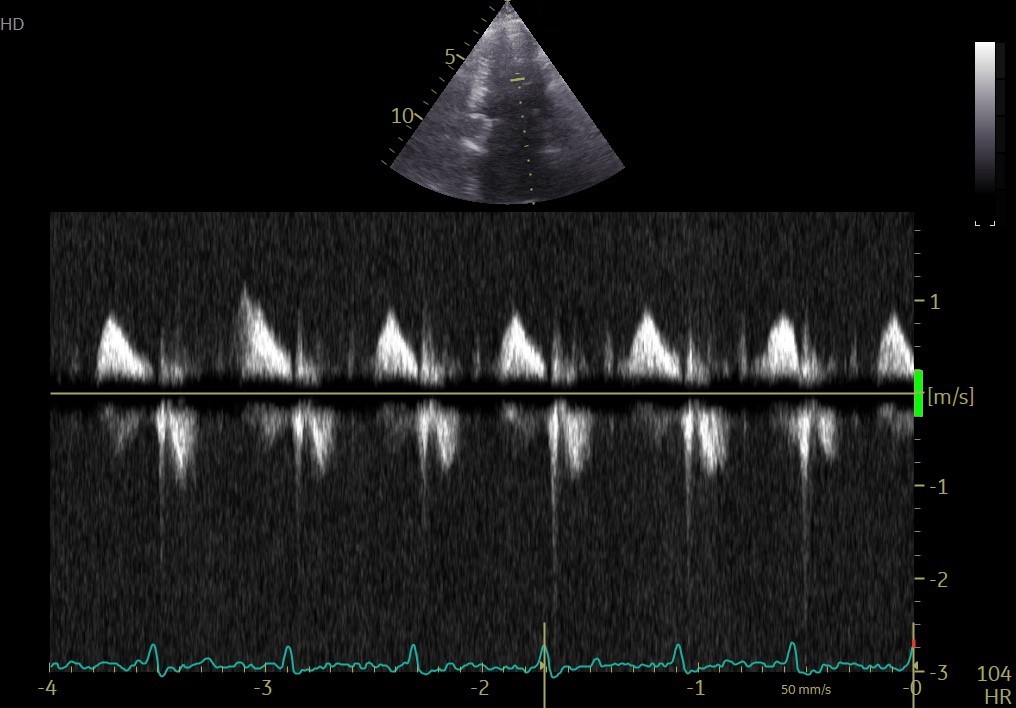

Supplement: ytae566_Supplementary_Data [file ytae566_supplementary_data.zip › CW STANDING.jpg]

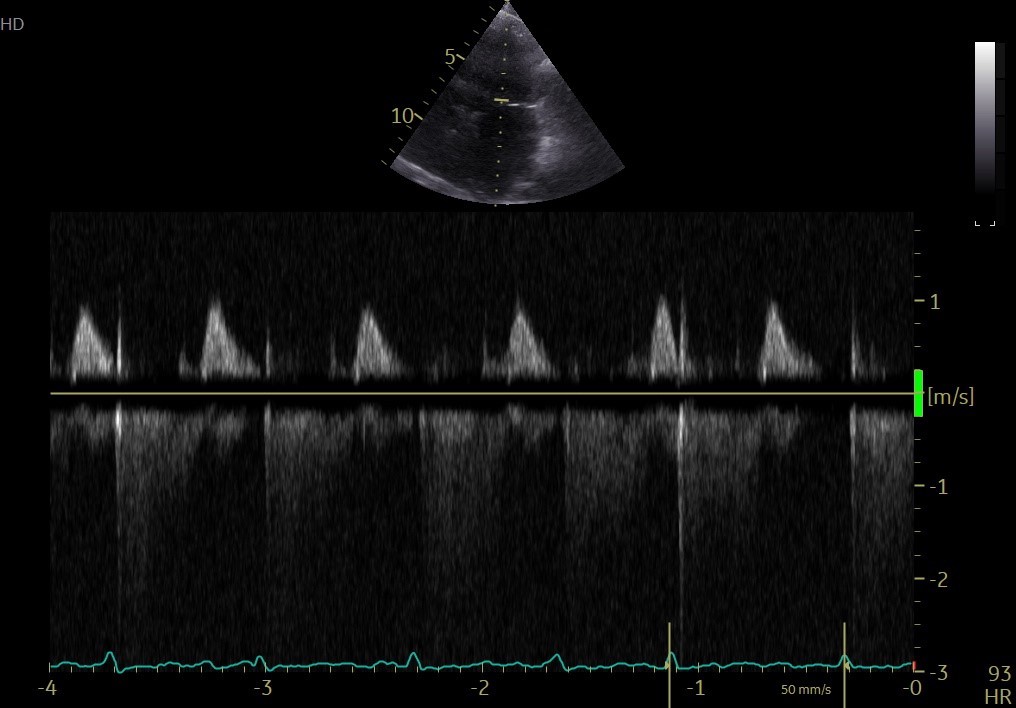

Supplement: ytae566_Supplementary_Data [file ytae566_supplementary_data.zip › Figure S1 supine.jpg]
